# Does the Differential Structure of Space-Time Follow from Physical Principles?

**DOI:** 10.3390/e26030179

**Published:** 2024-02-20

**Authors:** Rathindra Nath Sen

**Affiliations:** Department of Mathematics, Ben-Gurion University of the Negev, Beer Sheva 8410501, Israel; rsen@cs.bgu.ac.il

**Keywords:** relativistic causality, differentiable structures, uniform completion

## Abstract

This article examines Wigner’s view on the *unreasonable effectiveness of mathematics in the natural sciences*, which was based on Cantor’s claim that ‘mathematics is a free creation of the human mind’. It is contended that Cantor’s claim is not relevant to physics because it was based on his power set construction, which does not preserve neighborhoods of geometrical points. It is pointed out that the physical notion of Einstein causality can be defined on a countably infinite point set *M* with no predefined mathematical structure on it, and this definition endows *M* with a Tychonoff topology. Under Shirota’s theorem, *M* can therefore be embedded as a closed subspace of $R^{J}$ for some *J*. While this suggests that the differentiable structure of $R^{J}$ may follow from the principle of causality, the argument is constrained by the fact that the completion processes (analyzed here in some detail) required for the passage from $Q^{J}$ to $R^{J}$ remain empirically untestable.

## 1. Introduction

The title of this volume is ‘On the role of geometric and entropic arguments in physics’, and the title of this article could well be ‘On the role of physical arguments in geometry’, where geometry means differential geometry, the mathematical tool which made general relativity possible, and subsequently the theory of symmetry in quantum mechanics and gauge theories of elementary particle physics. Underlying these theories is the assumption that space-time is a differentiable manifold. Our question—which we shall try to answer—is the following: Is the differentiable structure of space-time an invention of mathematicians, or does it follow from *physical principles*, or to be precise, the principle of relativistic causality?

Simply asking this question represents a departure from the view that mathematics is invented and not discovered. Let us take a brief look—without pretensions to historical completeness—at how this view came to be articulated. For physicists, the marker is surely Wigner’s 1960 paper entitled ‘The unreasonable effectiveness of mathematics in the natural sciences’ [1]. As an answer to the question ‘What is mathematics?’, Wigner wrote the following: ‘… I would say that mathematics is the science of skillful operations with concepts and rules *invented* just for the purpose’ [emphasis added]. Mathematicians may trace it to Georg Cantor, founder of the theory of sets and transfinite numbers, who claimed in 1883 that ‘the very essence of mathematics is its freedom’ [2]. In citing this sentence, Fraenkel asserted in 1953 that ‘… we shall make plain the possibility of *free creation* in mathematics which is not equaled in any other science’ (p. 3 of [3]; the quotation from Cantor is on p. 4). In 1926, Hilbert had already declared that ‘No one shall expel us from the paradise which Cantor has created for us’ ([4], p. 170).

However, a year before the publication of Wigner’s paper, the Soviet geometer A. D. Alexandrov pointed out that the interiors of double cones (Note: By a *double cone*, we mean the nonempty intersection of a forward and backward light cone.) formed a base for the usual topology on Minkowski space [5]. In 1964, Zeeman published a paper with the title ‘Causality implies the Lorentz group’ [6]. The abstract of this paper reads as follows: ‘Causality is represented by a partial ordering on Minkowski space, and the group of all automorphisms that preserve this partial ordering is shown to be generated by the inhomogeneous Lorentz group and dilatations.’ These results suggest the possibility that *some* mathematical structures—here a Lie group structure—may be traceable to the laws of physics rather than the human imagination.

Then, in 1983, Donaldson announced a result, using gauge theory, which implied that the differentiable structure of $R^{4}$ was not unique [7]. (Note: The following notations are standard: R, QandZ denote the sets of real numbers, rational numbers and integers, respectively. There is some ambiguity with N, as some authors use it to denote the set of all *nonnegative* integers, which includes zero, and others use it to denote the set of positive integers. To avoid confusion, we shall denote the set of positive integers by N and the set of nonnegative integers by N.) In the same year, Gompf established that there were at least three inequivalent differentiable structures on $R^{4}$ [8]. Since $R^{4}$ is of particular interest to physicists, these results prompted the late H.-J. Borchers and the present author to undertake a systematic, *mathematical* investigation of the mathematical implications of relativistic causality. A full report of these investigations was published in 2006 as a volume in the Springer *Lecture Notes in Physics* series [9].

The end result of these investigations can be summed up as two theorems (omitting some fine points which can be ignored for the present without harm).

**Theorem** **1.** *A causal structure can be defined on a countably infinite point set M which has no predefined mathematical structure to it. This causal structure defines a Tychonoff $\left(T_{\frac{3}{2}}\right)$ topology on M and therefore a uniformity *(*called the* order uniformity**)* on it.*

The construction begins with defining distinguished, totally ordered subsets called *light rays* on *M* (In physical terms, these will be paths of light rays in space-time.). The total order on the light ray *l* is denoted by ${<}^{l}$. A crucial property of a light ray is the density property, where between any two distinct points *x* and *z* on a light ray *l* lies a third point *y*, and if $x,z\in l,\;x{<}^{l}z\;$ then there exists y∈l,y≠x,z such that $x{<}^{l}y{<}^{l}z$.

**Theorem** **2.** *The order uniformity can be uniformly completed and the order extended to the uniform completion. According to a theorem of Shirota, the completed space can be embedded as a closed subspace of some $R^{J}$, inheriting the differentiable structure of the latter.*

The mathematics used in the proof of Theorem 1 was developed ab initio; it does not use results from the existing corpus of mathematics. The proof of Theorem 2 uses a *completion* process which *creates* new points, as well as the fact that $R^{J}$ has a differential structure. Is the completion process a discovery or an invention? Is the differentiable structure of R a discovery or an invention?

The aim of this paper is to discuss these two questions, which are interesting, not strictly mathematical and (paraphrasing Bertrand Russell) may not have definite answers. They were not discussed in [9]. This discussion is carried out in the following section.

## 2. Why Is Mathematics Considered an Invention?

To answer this question, we have to recapitulate the basic concepts of set theory. We begin with Cantor’s definition of a set and his power set construction.

### 2.1. What Cantor Did

Cantor defined a set to be ‘a collection of different, distinct objects of our intuition or of our intellect, to be conceived of as a whole’ ([3], p. 6). Two sets were defined to be *equivalent* if they could be put in one-to-one correspondence with each other. For example the sets {1,2,3} and {♠,♡,♣} are equivalent, but the sets {1,2,3} and {>,<} are not. The property of equivalence divides the aggregate of finite sets into *equivalence classes*, with an equivalence class being characterized by the *number* of elements in a member of the set. (Here and in the following, we shall ignore some subtleties. This will not affect our argument materially.)

Using the definition of equivalence, Cantor gave a precise definition of an *infinite* set: a set is infinite if and only if it is equivalent to a *proper* subset of itself. For example, the set of positive integers is equivalent to the set of positive *even* integers, and the invertible map n↔2n establishes the equivalence. It is an easy exercise to show that the sets N (positive integers) and Z (all integers) are equivalent. Sets equivalent to N are called *countably  infinite* or simply *countable* or *enumerable*. It is only slightly more difficult to prove the counterintuitive result that the set Q of *rational numbers* is countable.

One of Cantor’s most important results was that some infinities are ‘bigger’ than others. The set of real numbers, which is infinite, is not countable. It will suffice to prove this for the reals values in the interval [0,1]. The proof is carried out by contradiction.

Assume that $a_{1},a_{2},\ldots$ is an enumeration of the real values in [0,1]. Then, $a_{k}$ can be expressed as

$$a_{k}=0.a_{k1}a_{k2}\ldots a_{kk}\ldots$$

where $a_{kn}$ is the $n^{\mathrm{th}}$ digit after the decimal point in the decimal expression for $a_{k}$. Now, we form the number

$$b=0.b_{1}b_{2}\ldots b_{n}\ldots$$

where

$$b_{j}=\left\{\begin{array}{ccc} a_{jj}+1, & \mathrm{if} & a_{jj}\neq 9\\ 0, & \mathrm{if} & a_{jj}=9 \end{array}\right.$$

Clearly, b∈[0,1]. This differs from $a_{j}$ in the *j*th digit after the decimal point, contradicting the assumption that all real values in [0,1] are contained in the enumeration $a_{1},a_{2},\ldots$ Note that there are many such numbers *b*. The (arbitrary) choice of $b_{j}=1\;\;\mathrm{if}\;\;a_{jj}\neq 1$ and $b_{j}=0\;\;\mathrm{if}\;\;a_{jj}=1$ works just as well. The sets N and R are both infinite, but R appears to be, in some sense, ‘bigger’ than N.

Equivalent finite sets have the same *number* of elements. Cantor generalized the notion of numbers to sets equivalent to N and R. He named this generalization *cardinality*, or *cardinal numbers*, and assigned the symbols ${ℵ}_{0}$ (from the Hebrew letter *ℵ*, pronounced ‘aleph’) to the cardinality of N and **c** to that of R. (Note: Fraenkel [3] used *ℵ* for the cardinality of R, reserving c for the generic cardinal. Hausdorff in his classic text [10] also used *ℵ* for the cardinality of R.) The cardinality of a finite set is, of course, the number of elements in the set.

Now we come to the critical point: Cantor’s power set construction. According to Cantor’s definition of a set, given *any* set *S*, its *power set*, defined as the *set of subsets* of *S* and denoted by P(S) or $2^{S}$, is a well-defined set. He showed that *the cardinality of $2^{S}$ was always greater than that of S*. Thus, by starting with a set *S* of cardinality ${ℵ}_{0}$ and iterating the power set construction, one obtains a non-terminating sequence of sets P(S),P(P(S))… of increasing cardinality, which were labeled

$${ℵ}_{1},{ℵ}_{2},{ℵ}_{3},\ldots {ℵ}_{n}\ldots$$

by Cantor. The alephs were called *transfinite cardinals* by him. The power set construction displayed the power of ‘free creation’ in mathematics.

### 2.2. Russell’s Paradox and Its Resolution

In 1901, Bertrand Russell discovered that Cantor’s definition of a set was so general as to be unsustainable. (A good elementary account with references can be found in [11].) Sets are usually not members of themselves. The set *R* of all physicists is definitely not a physicist, and therefore R∉R. According to Cantor, one may define the set *C* of *all* sets which are *not* members of themselves. Then, *C* is a member of *C* (C∈C), meaning that *C* is *not* a member of *C* (C∉C), which is a contradiction!

Mathematicians quickly realized that Cantor’s notion of sets was too general, and some restrictions had to be put in place. The axiom system (for sets) which gained the greatest acceptance was set up by Zermelo and Fraenkel, and it is known as *ZF* [12]. The power set construction and Cantor’s theory of transfinite numbers remain valid in *ZF*. Mathematicians generally work with *ZFC*, where *C* stands for the independent *axiom of choice*.

The conjecture is that ${ℵ}_{1}=c$ is known as the *continuum hypothesis*. Cantor tried in vain for the rest of his life to prove the continuum hypothesis. In 1940, Kurt Gödel showed that ${ℵ}_{1}\neq c$ could not be proven in *ZFC*, and in 1963, Paul Cohen showed the same was true of ${ℵ}_{1}=c$. Both the continuum hypothesis and its negation were consistent with *ZFC*.

### 2.3. Physics and the Power Set Construction

Early in his book, Fraenkel asserted that ‘… we shall make plain that possibility of *free creation* in mathematics which is not equaled in any other science’ ([3], p. 3). This assertion clearly pointed to the power set construction on which the theory of transfinite numbers is based, but how relevant is this construction to physics? We claim that the power set construction does not produce physically meaningful sets when applied to space-time manifolds, which are the most important uncountably infinite sets used in physics.

In physics, the notion of a geometrical point is an idealization. The mere recording of the infinitely many digits of an irrational number will demand infinite physical resources and infinite time. The position of a point in space can only be determined approximately. From the empirical point of view, the important notion is the slightly fuzzy one of the *neighborhood* of a point. While the notion of the *continuum* is not directly accessible to experimentation, it is the foundation on which the notions of differentiable and analytic functions are based, and these notions are the basic tools of discovery of the theoretical physicist. The predictive power of general relativity would not be diminished if its differential equations were to be replaced by difference equations, but could they have been discovered in the difference equation form?

In brief, in physics one can *never* separate a geometrical point from its neighborhood. (Note: To be precise, here *neighborhood* is taken to mean neighborhood in the standard topology of space-time.) But that is *exactly* what the power set construction does; it destroys the essential, necessarily fuzzy notion of the neighborhood when applied to *any* continuum!

### 2.4. The Mathematics Needed by Physics

For this reason, the present author contends that the mathematics which derives from application of the power set construction to infinite sets is not relevant to physics. The only infinities which physics needs are the integers and rational and real numbers (the continuum). It may be useful to know that their cardinalities ${ℵ}_{0}$ and c are different, but the continuum hypothesis has no relevance.

The moment one drops the power set construction (for infinite sets), one loses the raison d’être for the contention that mathematics is an invention of the human mind.

## 3. From the Discrete to the Continuous

As was already pointed out, the mathematics used for establishing Theorem 1 was developed ab initio. This is no longer the case with Theorem 2.

The mathematics used to establish Theorem 2 is, first and foremost, the process called *completion* in mathematical analysis. It would suffice to consider *metric completion*, the archetype of which is the completion of the rational numbers Q to the real numbers R under the metric d(x,y)=|x−y|,x,y∈Q.

To explain the process, we need to recall the following well-known definitions and examples:

**Definition** **1.** *A sequence $\left\{a_{n},n\in N\right\}$ of real numbers is called a *Cauchy sequence* if, given *any* ϵ>0, there exists a positive integer N such that*

$$|a_{k}-a_{l}|<\epsilon \;\;\mathrm{for}\;\mathrm{all}\;\;k,l>N$$

**Example** **1.** *Consider the sequence $\left\{s_{n}=1/n|n\in N\right\}$. This is a Cauchy sequence, because for any ϵ>0, there exists a positive integer N such that 1/N<ϵ. Then, for m,n>N, we have 1/m,1/n>ϵ such that*

$$|s_{m}-s_{n}|=\left(\frac{1}{m}-\frac{1}{n}\right)<\epsilon$$

**Definition** **2.** *Let S be a subset of R. A sequence $\left\{s_{n}\right\}$ in S is said to *converge* to s if there exists s∈S such that, given ϵ, there exists N such that $|s_{n}-s|<\epsilon$ for all n>N.*

The sequence $\left\{s_{n}=1/n|n\in N\right\}$ converges to 0 *in any interval* (x,y) which contains the point 0. It *does not converge* in intervals (a,0) and (0,b), which *do not contain* the point 0.

We need one last definition, namely that of *completeness*:

**Definition** **3.** *A metric space S (with metric d(x,y)) is said to be *complete* if every Cauchy sequence in S converges (to a point in S).*

For uniform spaces, (A *uniform structure* in a space is weaker than a metric structure, but it is a space which admits a differentiable structure [13].) the notion of completeness still holds. One has to only replace the sequences with filters.

**Example** **2.** *The space of real numbers R, with the metric d(x,y)=|x−y|, is complete. The space of rational numbers Q (with the same metric) is not complete. All closed intervals [a,b] are complete, and all open intervals (a,b) are incomplete.*

**Example** **3.** *Consider the sequence $\left\{x_{n}|x_{n}\in Q,n\in N\right\}$ defined by*

(1) $$x_{1}=1,\;x_{n+1}=\frac{1+\frac{2}{x_{n}}}{2}$$

*It can be shown, with some effort, that the sequence $\left\{x_{n}\right\}$ defined by Equation (1) is a Cauchy sequence which converges to $\sqrt{2}$. It converges in R but not in Q (since $\sqrt{2}$ is irrational).*

This algorithm of approaching $\sqrt{2}$ with successive approximations (calculating $x_{n+1}$ from $x_{n}$) was known in several ancient civilizations.

The process of *completing* Q to R—passing from the discrete to the continuous—is exemplified perfectly by the simple example described above, which *defines the irrational number $\sqrt{2}$ with a Cauchy sequence of rationals, namely the sequence $\left\{x_{n}\right\}$*. More generally, in a complete metric space, every Cauchy sequence (Cauchy *filter* in complete non-metric Tychonoff spaces) converges, and one *completes* an incomplete space by defining new points with Cauchy sequences which do not converge in the original space. Every point in the complete space may equally be regarded as a Cauchy sequence. In the completed space, all Cauchy sequences converge.

## 4. Putting the Pieces Together

Assuming we have agreed that the power set construction is irrelevant to physics, the two questions raised at the end of Section 1 remain to be answered. Is the continuum (space-time, for example) a discovery or an invention? In other words, is the convergence of every Cauchy sequence in space-time a fact of nature or merely a mathematical artifice? Is the differentiable structure of $R^{N}$ a discovery or an invention?

### 4.1. Is the Continuum a Discovery or an Invention?

The totality of the experimental data can be represented as a finite set of *n* tuples of rational numbers. The convergence or divergence of an infinite series depends on its infinite ‘tail’ and not on its finite ‘head’. It is not possible to determine through experimentation (rather, observation) whether or not an arbitrary sequence of numbers is a Cauchy sequence for the simple reason that it is impossible to write down explicitly *all* the terms of an infinite sequence on a piece of paper.

The laws of physics—classical and quantum mechanics and electrodynamics, relativity theory as well as most forms of quantum field theory—are based on the assumption that space, time and space-time are continua. However, this assumption may no longer be tenable if Einstein’s theory of gravitation turns out to be quantizable. Although these attempts have not succeeded thus far, they are still continuing in the form of string theory. The present situation may therefore be summed-up as follows.

Mathematical continua are well-defined entities. Whether or not they are identifiable with physical space, time or space-time has not yet been settled. An affirmative answer—which, in the present state of knowledge, would require the possibility of examining infinite sequences on a blackboard—seems to be out of reach. A negative answer may be possible if gravity can be quantized successfully.

### 4.2. Is the Differentiable Structure of $R^{N}$ a Discovery or an Invention?

Given the one-dimensional mathematical continuum R, one may argue that differentiable functions on it are provided ‘by nature’, and no human artifice like the completion process is needed; that is, the differentiable structure of R, and likewise that of $R^{N}$, is a discovery and not an invention. This would seem to be reinforced by the existence of ‘exotic’ differentiable structures on $R^{4}$. On the other hand, if the continuum itself is a human invention, it is surely legitimate to ask how nature can provide mathematical structures on objects which do not ‘exist’ in nature.

It appears that the question posed in the title of this paper may well be unanswerable. It is, however, surprising how far the principle of relativistic causality has led us toward an answer.

## Data Availability

No new data were created or analyzed in this study.
